# Cognitive trajectories in multiple sclerosis: a long-term follow-up study

**DOI:** 10.1007/s10072-021-05356-2

**Published:** 2021-06-08

**Authors:** Antonio Carotenuto, Teresa Costabile, Giuseppe Pontillo, Moccia Moccia, Fabrizia Falco, Maria Petracca, Martina Petruzzo, Cinzia Valeria Russo, Martina Di Stasi, Chiara Paolella, Teresa Perillo, Elena Augusta Vola, Maria Brunella Cipullo, Sirio Cocozza, Roberta Lanzillo, Vincenzo Brescia Morra, Francesco Saccà

**Affiliations:** 1 Department of Neurosciences, Reproductive and Odontostomatological Sciences, Federico II University, Naples, Italy; 2 Department of Advanced Biomedical Sciences, Federico II University, Naples, Italy

**Keywords:** Cognitive dysfunction, Hippocampus, Biomarkers, Magnetic resonance imaging, Disability predictors, Longitudinal data analysis

## Abstract

**Background:**

Cognitive impairment occurs in multiple sclerosis (MS) and undergoes a progressive worsening over disease course. However, clinicians still struggle to predict the course of cognitive function. To evaluate baseline clinical and imaging predictors of cognitive abilities worsening over time, we performed a latent trajectory analysis for cognitive performances in MS patients, up to 15 years from disease onset.

**Methods:**

We collected age, sex, education, dominant and non-dominant 9-hole peg test (9HP) and timed 25-foot walk (T25-FW) as well as MRI measures (grey matter volume and lesion load) within 6 months from disease diagnosis for relapsing–remitting MS (RR-MS) patients. At diagnosis and over the follow-up, we also assessed cognitive status through the symbol digit modalities test (SDMT). Cognitive impairment was defined by applying age-, gender- and education-adjusted normative values. Group-based trajectory analysis was performed to determine trajectories, and the predictive value of clinical and imaging variables at baseline was assessed through multinomial logistic regression.

**Results:**

We included 148 RR-MS (98 females and 50 males). Over 11 ± 4 year follow-up, 51.4% remained cognitively stable whereas 48.6% cognitively worsened. Cognitively worsening patients had a higher T25FW time (*p* = 0.004) and a reduced hippocampal volume at baseline (*p* = 0.04).

**Conclusion:**

Physical disability as well as hippocampal atrophy might depict patients at risk of cognitive worsening over the disease course. Therefore, using such predictors, clinicians may select patients to carefully evaluate for cognitive impairment as to eventually introduce cognitive rehabilitation treatments.

## Introduction

Cognition can be impaired in up to 70% multiple sclerosis (MS) patients from disease onset [1], mostly affecting attention, information processing speed and long-term memory [2] with a negative impact on everyday activities [3]. Lesion load and global and regional brain atrophy, together with microstructural changes throughout the brain, are associated with deficits in cognition for MS patients in cross-sectional studies [4, 5]. Over the disease course, cognitive function might further worsen [6]. However, clinicians still struggle to predict whether a patient will deteriorate in terms of cognitive abilities over the long-term follow-up due to the lack of baseline reliable predictive biomarkers. Available longitudinal studies showed that increased lesion load and grey and white matter atrophy are associated with cognitive worsening in MS [7–11]. Baseline measures such as grey matter volume, temporal atrophy, diffuse microstructural changes and, specifically, microstructural alterations in the anterior thalamic radiations and the superior longitudinal fasciculus might predict cognitive outcome in MS up to 7 years from disease onset [11, 12]. However, in order to better tailor disease management and treatment strategies at disease diagnosis, taking into account also the possibility of cognitive deterioration over time with subsequent negative impact on social life, predictive biomarkers for long-term cognitive outcome in MS are extremely needed.

Group-based trajectory analysis is a statistical approach allowing to identify and summarize complex patterns in longitudinal data assuming that the sample is composed of a mixture of different groups following similar longitudinal trends for a specific variable [13]. Initially, this approach was applied in psychology [14] and criminology [15], but only recently it was applied also in medicine, especially for evaluating cognitive trajectories in people with mild cognitive impairment [16, 17]. Hereby, we aim at classifying long-term longitudinal cognitive trajectories of MS patients using the group-based trajectories analysis. Moreover, we also aim to evaluate the demographic, clinical and imaging measures predicting cognitive worsening over long-term follow-up.

## Methods

### Study design

This is a retrospective study including newly diagnosed relapsing–remitting MS subjects (RR-MS), with cognitive assessment at diagnosis that have been followed up prospectively up to 15 years.

### Subject enrolment

We included subjects receiving a new diagnosis of RR-MS from January 2004 to January 2012 at the ‘Federico II’ University (Naples, Italy).

Inclusion criteria were (i) diagnosis of RR-MS according to 2001, 2006 or 2010 McDonald’s criteria as appropriate [18–20] and (ii) ≤ 6 months from disease diagnosis. Exclusion criteria were past or present systemic medical conditions or psychiatric diseases or treatments that might impact on cognitive performances (i.e. chemotherapy, or corticosteroid treatment from less than 1 month).

Exclusion criteria were past or present systemic medical conditions or psychiatric diseases.

Sociodemographic (age, gender and education) and clinical data (age at onset, disease duration, EDSS, 9-hole peg board test for dominant [9HP-D] and non-dominant hand [9HP-ND] and Timed 25-Foot Walk [T25-FW], global annualized relapse rate calculated as total number of relapse before enrolment over disease duration from disease onset) were collected at baseline. MS subjects were treated with different disease-modifying treatments (DMTs), possibly changed or discontinued during the study period as for clinical practice. DMTs including interferon-beta, glatiramer acetate, dimethylfumarate and teriflunomide were classified as first line, while all other DMTs were classified as second-line DMTs. Changes in DMTs, the occurrence of phenotype conversion from RR-MS to secondary progressive MS (SPMS), following clinical diagnosis, and the occurrence of relapses over the follow-up were recorded. Follow-up visits were scheduled as for clinical practice (mostly every 6 months or on the occasion of relapses).

### Neuropsychological assessments

Cognitive function at baseline was assessed within 6 months from disease onset through the Symbol Digit Modalities Test (SDMT) [21]. SDMT rough scores were age, gender and education corrected by applying the available normative values and cognitive impairment was defined if the corrected scores fell below the 5th percentile [22, 23]. Only patients with at least three different cognitive assessments (including the baseline assessment) were enrolled in the study.

### MRI data acquisition and analysis

MRI scans were acquired at baseline on 3 T scanner (Trio, Siemens Medical Systems, Erlangen, Germany), with the acquisition protocol including a 3D T1-weighted magnetization-prepared rapid acquisition gradient echo sequence (MPRAGE; TR = 1900 ms; TR = 3.39 ms; TE = 3.4 ms; TI = 900 ms; flip angle = 9°; voxel size = 1 × 1 × 1 mm^3^; field of view = 250 × 250; 160 axial slices) and a fluid-attenuated inversion recovery sequence (FLAIR; TR = 9620 ms; TE = 30 ms; TI = 2500 ms; field of view = 250 × 250; voxel size = 1 × 1 × 3 mm^3^; 48 axial slices).

For the determination of brain volume measurements, hyperintense lesions on FLAIR images were identified and segmented using a semiautomatic approach (Jim 7, Xinapse Systems, Northants, UK). Then, to correct for the possible impact of white matter (WM) lesions on volume measurements, the resulting lesion masks were co-registered via affine registration to the MPRAGE, and masked-out. From lesion masked MPRAGE images, grey matter (GM) and normal appearing WM (NAWM) volumes were estimated with FSL’s Structural Image Evaluation Using Normalization of Atrophy (SIENAX) [24], and the normalized brain volume (NBV) was calculated summing GM and WM volumes and multiplying such value for the SIENAX scaling factor. Furthermore, subcortical GM volumes were also obtained, using the FIRST routine (FMRIB’s Integrated Registration Segmentation Toolkit http://fsl.fmrib.ox.ac.uk/fsl/fslwiki/FIRST) [25], obtaining left and right caudate nucleus, pallidum, putamen, thalamus and hippocampus volumes, which were subsequently averaged. Similarly to the procedure done with the NBV calculation, deep GM volumes were also normalized by multiplying for the SIENAX scaling factor.

### Statistical analyses

Statistical analyses were performed using Stata software (version 13; StataCorp LP, College Station, TX). Demographic, clinical and cognitive features of MS patients were presented as means, medians or proportions as appropriate. Group-based trajectory analysis was carried out using the SAS procedure Proc Traj embedded in STATA [26]. Group-based trajectory analysis identifies latent subgroups from a larger sample with distinct trajectories over time for the outcome measures. Time can vary by person and subjects will be assigned to a specific group based on the largest posterior probability of group membership. Considering MS phenotype and presumptive disease burden over time, models with 2–5 latent subgroups were assessed and the optimal number of subgroups for each outcome measure was defined by selecting the lowest Akaike’s information criterion (AIC) [27]. In order to evaluate longitudinal trajectories, we performed a group-based trajectory analysis using a regression model, including SDMT-corrected scores at each time point as dependent variable, disease duration calculated from disease onset as an independent variable, gender, age at onset and annualized relapse rate at diagnosis and EDSS at baseline were examined as time-stable covariates and disease-modifying treatment and the occurrence of relapses were used as time-varying covariates, thus correcting the models for such changes and evaluating their impact on the overall trajectories.

Once membership was assigned, we assessed the predictive value of clinical (9HP-D, 9HP-ND, T25FW and the occurrence of conversion to SPMS) and imaging (normalized cortical grey matter, T2 lesion load, caudate nucleus, pallidum, putamen, thalamus and hippocampus volume) variables at baseline using an age- and gender-adjusted logistic regression. Odds ratio (OR) was reported. We evaluated normal distribution of variables and/or residuals through statistical and graphical approaches. The *p* value was not corrected for multiple comparisons due to the exploratory nature of the analysis. Results were considered statistically significant for *p* < 0.05.

## Results

### Baseline features

We included 148 RR-MS patients (98 females and 50 males). Patients were followed up to 11 ± 4 years. Sixty patients underwent MRI scan at baseline. Demographic, clinical and neuroimaging data at enrolment are summarized in Table 1.

**Table 1:** Demographic, clinical and neuroimaging features at baseline

Characteristic		
Subjects	Total	148
Sex	Male, N (%)	50 (34)
	Female, N (%)	98 (66)
Age, mean ± SD (range) (years)		35.1 ± 8 (16–58)
Age at diagnosis, mean ± SD (range) (years)		28.4 ± 7.5 (13–45)
Disease duration from onset, mean ± SD (range) (years)		6.7 ± 0.1 (0.1–30.9)
EDSS, median (range)		2 (1.5–4.5)
ARR, mean ± SD (range)		0.94 ± 0.41 (0–2.5)
Timed 25-foot walk, mean ± SD (range)		5.9 ± 0.2 (5.6–6.3)
Nine hole peg-board test dominant hand, mean ± SD (range)		20.7 ± 0.5 (19.8–21.6)
Nine hole peg-board test non dominant hand, mean ± SD (range)		21.8 ± 0.5 (20.7–22.8)
Follow-up time, mean ± SD (Range) (years)		11 ± 4 (1–35)
Number of cognitive assessment over the follow-up, median (range)		4 (2–5)
Cortical grey matter (volume), mean ± SD		756.2 ± 50.92
Normal appearing white matter (volume), mean ± SD		667.8 ± 42.92
T2-weighted lesion load (volume), mean ± SD		7.3 ± 7.92
Caudate nucleus (volume), mean ± SD		3.6 ± 0.59
Pallidum (volume), mean ± SD		1.7 ± 0.22
Putamen (volume), mean ± SD		4.9 ± 0.76
Thalamus (volume), mean ± SD		7.3 ± 0.9
Hippocampus (volume), mean ± SD		3.9 ± 0.5

*ARR* annualized relapse rate; *N* number; *EDSS* expanded disability status scale; *SD* standard deviation. All volumes are expressed in milliliters

At baseline, 19 out of 148 patients (12.8%) showed a deficit in SDMT (Table 2). After about 10 years, 36 out of 148 patients (24.3%) showed a deficit in SDMT.

**Table 2:** Clinical and cognitive assessments over the follow-up

Time points		1	2	3	4	5	6
Number of subjects		148	148	148	125	76	33
Time from baseline cognitive assessment, mean ± SD (years)		-	1.98 ± 0.21	9.96 ± 1.92	11 ± 1.89	11.42 ± 1.76	11.93 ± 1.51
Time from previous cognitive assessment, mean ± SD (years)		-	1.98 ± 0.21	7.96 ± 1.92	1.12 ± 0.34	1.09 ± 0.25	1.04 ± 0.19
EDSS, mean ± SD		2.3 ± 0.7	2.5 ± 0.8	3.3 ± 1.1	3.4 ± 1.3	3.6 ± 1.6	3.6 ± 1.4
% of patients experiencing a relapse		-	33.1	59.2	4.8	3.4	0.7
Disease-modifying therapy	No treatment, (%)	62	1.4	2	0.80	1.3	0
	First line, (%)	38	97.2	76.2	62.4	44.8	42.4
	Second line, (%)	0	1.4	21.8	36.8	53.9	57.6
SDMT, mean ± SD		50.5 ± 12.7	45.8 ± 12.1	43.9 ± 12	43.7 ± 11.9	43.6 ± 11	44.3 ± 11
% of patients failing the test		12.8	25	23.8	24	23.4	27.3

*EDSS* expanded disability status scale; *SD* standard deviation; *SDMT* symbol digit modalities test

### Cognitive function trajectories

**Fig. 1:**
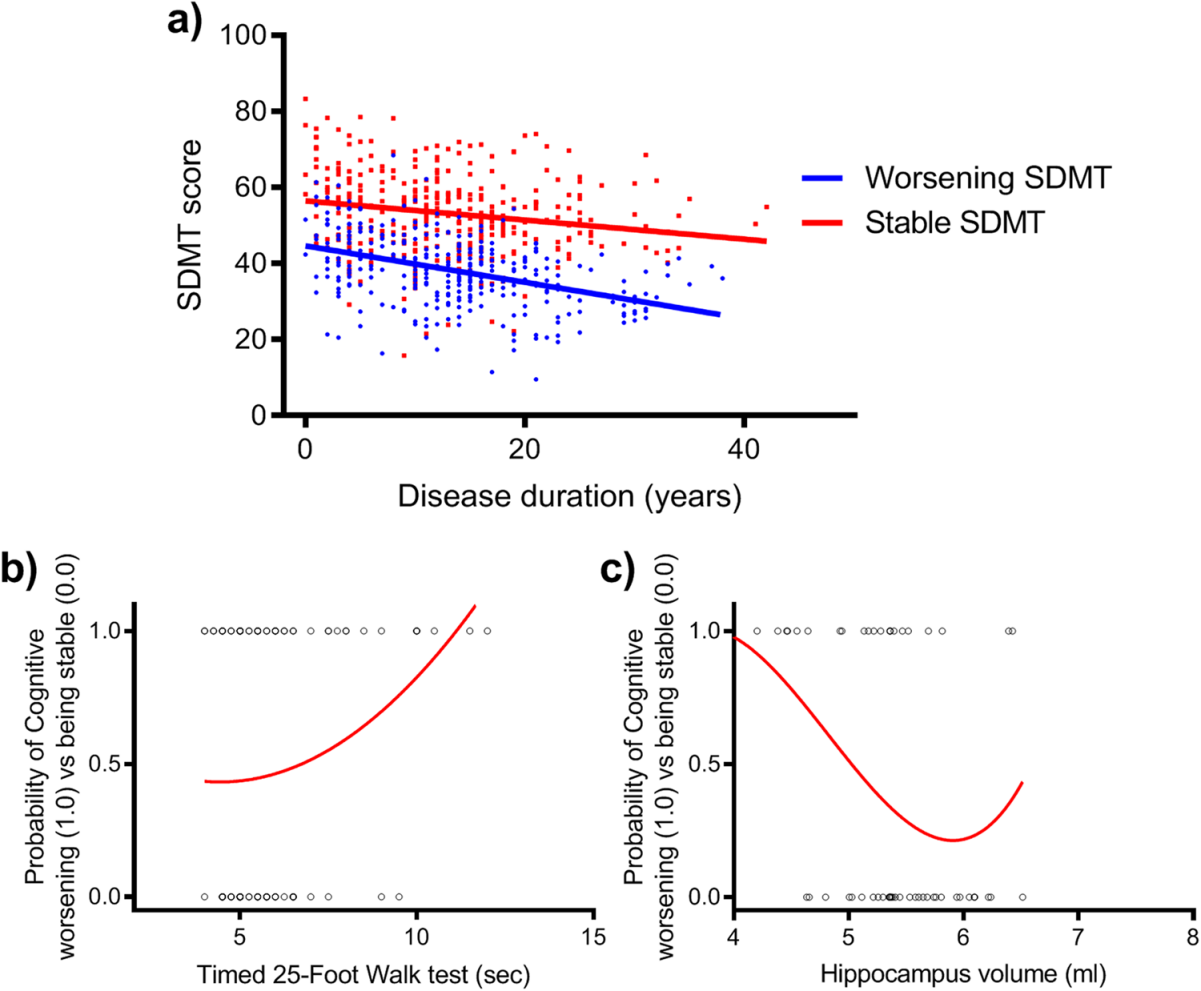
Cognitive trajectories based on SDMT score from baseline over the follow-up and predictors of cognitive abilities worsening. **a** We identified 2 cognitive trajectories based on SDMT score: MS patients without SDMT worsening over time (76 patients, 51.4%) or who developed SDMT worsening over time (72 patients, 48.6%). The probability of belonging to SDMT stable group increases with the **b** shorter timed 25-foot walk (*p* = 0.004) and **c** higher hippocampal volume (*p* = 0.04)

By comparing the AIC coefficient, we selected the 2 trajectories’ model. We depicted MS patients with stable SDMT (76 patients, 51.4%) and MS patients experiencing SDMT worsening over time (72 patients, 48.6%). SDMT trajectories are reported in Fig. 1a. Amid patients classified as cognitively stable, only 2 out of 76 (3%) showed a SDMT deficit, while for patients classified as cognitively worsened, 34 out of 72 (47.2%) were classified as impaired.

Cognitive worsening over time was predicted by T25FW and hippocampal volume (OR: 0.25; 95%CI = 0.10, 0.64; *p* = 0.004; and OR: 5.1; 95%CI = 1.12, 22.8; *p* = 0.04, respectively; Fig. 1b, c; Table 3).

**Table 3:** Baseline clinical and imaging predictors of cognitive worsening over time

Group	Cognitively stable patients	Cognitive-declining patients	OR	95% CI	*p* value*
Number of patients	76	72			
9-hole peg test, dominant hand, mean ± SD (seconds)	19.08 ± 2.79	22.31 ± 5.89	0.72	0.5—1.05	0.19
9-hole peg test, non-dominant hand, mean ± SD (seconds)	20.3 ± 3.87	23.35 ± 6	1.19	0.88—1.6	0.29
Timed 25-foot walk, mean ± SD (seconds)	5.53 ± 1.07	6.36 ± 2.04	0.25	0.10—0.64	0.004*
Normalized cortical grey matter, mean ± SD (ml)	767.26 ± 49.73	742.61 ± 49.93	0.95	0.86—1.05	0.45
T2 lesion load, mean ± SD (ml)	8.38 ± 8.22	12.3 ± 13.79	1.01	0.99—1.03	0.12
Caudate nucleus volume, mean ± SD (ml)	4.92 ± 0.57	5.02 ± 0.79	0.9	0.53—1.07	0.09
Pallidum volume, mean ± SD (ml)	2.42 ± 0.22	2.34 ± 0.31	3.2	0.04—282.12	0.61
Putamen volume, mean ± SD (ml)	6.84 ± 0.66	6.7 ± 1.14	0.25	0.06—1.11	0.07
Thalamus volume, mean ± SD (ml)	10.3 ± 0.93	9.72 ± 1.28	0.78	0.2—3.07	0.72
Hippocampus volume, mean ± SD (ml)	5.53 ± 0.46	5.24 ± 0.84	5.1	1.12—22.8	0.035*

*OR* odds ratio; *CI* confidence intervals; *SD* standard deviation

^*^Age- and gender-adjusted logistic regression

## Discussion

In this retrospective study, we investigated cognitive trajectories in a large cohort of RR-MS patients over a long-term follow-up (up to 15 years). We observed that the cognitive function in MS might either remain stable or deteriorate over time. We also pointed out that patients undergoing cognitive worsening over long-term follow-up show a higher degree of motor disability as well as reduced hippocampal volume.

The major novelty of the present study is the data-driven approach to define trajectories based on longitudinal performance, identifying clusters of individuals who have followed a similar developmental trajectory on an outcome of interest [13, 28]. Differently from other available statistical approaches such as hierarchical modelling and latent curve analysis estimating the population average trajectory, group-based trajectory analysis assumes that the population is made of distinct groups, each one following a different underlying trajectory. Group-based trajectory analysis identifies groups of individuals following similar progressions of some phenomenon over time taking into account the effects of covariates on both trajectory shape and group membership throughout the follow-up period for each single subject. Particularly, time-stable covariates influence group membership, while time-dependent covariates explain variation about the average trajectory within each group. For these characteristics, group-based trajectory modelling should be preferred to hierarchical and latent curve modelling when handling non-monotonic trajectories and trajectories that do not vary regularly in the population. Moreover, while raw categorization in stable versus worsening patients only considers the first and last measure, here, we took advantage from the trajectory analysis, providing the unique opportunity to depict the trend for each measure throughout the time span. This might be particularly relevant in disorder such as MS, where fluctuating symptoms like fatigue, anxiety or depression may harshly impact cognitive performance [29]. The importance of such approach was also recently highlighted from Healy et al. who were able to classify patients with SDMT worsening over time using a latent trajectory analysis adjusted for demographic covariates [30]. However, authors did not seek to evaluate baseline predictors of SDMT worsening over time. With this study, we evaluated trajectories for SDMT and we also sought to investigate baseline predictors of cognitive decline. Previous studies report that the proportion of MS patients showing cognitive decline spans from 28 to 70% [11, 12, 31, 32]. This wide range is mostly due to the number tests/domains explored and also to the different definitions of cognitive decline. Here, we found that 48.7% of MS patients deteriorated over a long-term follow-up. Although this proportion is in line with previous findings, when considering our longer follow-up, one would expect a higher rate of cognitive worsening. In addition, we should also consider that amid these worsening patients were those contributing the most to SDMT impairment prevalence at follow-up, thus suggesting that trajectories provide a framework of cognitive longitudinal assessment encompassing pre-fixed time points.

Another noteworthy finding of the present study is that we highlighted distinct baseline clinical and radiological features able to predict the overall cognitive outcome. We observed that clinical measures of physical disability evaluating the walking speed is a strong predictors of cognitive decline. Benedict et al. already reported a close interplay between motor abilities and the information processing speed cognitive function in MS patients [33], and speculated that motor functions also depend upon intact cognition since the planning and the performance of the action require cognitive abilities such as attention and executive function [33]. The close interplay between motor abilities and cognitive functions in MS might depend upon the disruption of shared cortico-subcortical networks and cortical pathology. We might hypothesize that reduced walking speed at disease onset in patients developing cognitive impairment at follow-up reflects the presence of tissue microstructural changes and functional network disruption. This damage at baseline may not foster cognitive impairment, but predicts decline over time in cognitive functions, especially for information processing speed. Supporting this point, we also highlighted that hippocampal atrophy at baseline is associated with cognitive decline. Atrophy of the temporal lobe was already associated with cognitive decline in MS patients [12]. Recently, Eijlers and colleagues reported that cognitive decline was predicted by white matter integrity and deep grey matter volume in early stages of MS, whereas cortical atrophy leads to cognitive decline in more advanced MS patients or progressive MS [12]. A study conducted from the same study group pointed out that while cognitive decline is strongly associated with cortical neurodegeneration in progressive stages of the disorder and with deep grey matter atrophy in RR-MS patients converting to progressive stages, white matter damage leads to a slower cognitive worsening in stable RR-MS patients [34]. In our sample, we demonstrated that RR-MS patients, who will develop cognitive impairment over the long-term follow-up, already show reduced hippocampal volume. Previous studies reported an association between hippocampal atrophy and cognitive impairment in cross-sectional studies [35, 36]. Indeed, the fact that declining patients display atrophy even in the absence of cognitive impairment at baseline might suggest that MRI measures we adopted actually depict an ongoing pathological processes that will only subsequently impact clinically relevant measures such as cognitive impairment. One possible missing piece of the puzzle generating this clinical-radiological paradox might be the patients’ cognitive reserve, which is the ability of the brain to cope with damages compensatory mechanisms not necessarily related with brain volume but also with brain functioning. Cognitive reserve strongly depends, among the other factors, upon patient’s lifestyle, education and social activities [37]. In light with this finding, it is worth exploring the impact of cognitive rehabilitation treatment on cognitive outcome, as several reports have confirmed the efficacy of these treatments on cognitive symptoms [38, 39]. These treatments may either be based on training exercises to improve specific cortical functions or may be delivered through non-invasive brain stimulation aimed at foster brain activity over specific cortical and subcortical grey matter regions [40].

We do acknowledge that this study is not without limitation. Firstly, the lack of a control group prevents us to draw any final conclusion about the disease-specific cognitive worsening. We are aware that cognitive scores may reduce over the follow-up due to aging. However, in the attempt to overcome this issue, we did use the normative age- and gender-corrected scores. Similarly, the lack of the control group may also affect MRI results interpretation. Therefore, future longitudinal studies including HC group are highly recommended. Secondly, also depression and fatigue may impact on cognitive scores. The lack of such measures did not allow us to correct cognitive scores for neuropsychiatric features.

In conclusion, physical disability as assessed through T25FW as well as MRI measures might depict patients at risk of cognitive decline. Since cognitive impairment correlates with neurodegeneration and harshly affects patient’s quality of life, proxies for cognitive deterioration over time should lead clinicians to tailored treatment choices and rehabilitation planning. However, further longitudinal long-term follow-up studies aiming at evaluating not only cognitive but also brain atrophy trajectories could better elucidate the underlying interplay between cognitive reserve, brain pathology and both physical and cognitive disability accrual.

## Data Availability

Data will be made available upon reasonable request to the corresponding author.
